# Supplementary material for: A set of multi-entry identification keys to African frugivorous flies (Diptera, Tephritidae)
Source: Zookeys. 2014 Jul 24;(428):97–108. doi: 10.3897/zookeys.428.7366 (PMC4143993; doi:10.3897/zookeys.428.7366)
Supplement: Supplementary material 4 — Key to Capparimyia [file zookeys-428-097-s004.zip › SF4_ZooKeys_key to Capparimyia/lucid_player/help/help/whgdata/whlsti0.htm]

Index words list


**About Lucid family of products**  
    **Lucid3 Builder**  
**About Lucid3 player**  
**Activation**  
**All States**  
Allow Misinterpretations  
   **Setting up the Player**  
   **The Lucid Player’s Menu and Button Bar**  
Applet Player  
   **Help for the Lucid3 Applet Player**  
   **The Applet Player and Application Player**  
Application Player  
   **Registration and Activation**  
   **The Applet Player and Application Player**  
Automating  
   **Setting up the Player**  
   **The Lucid Player’s Menu and Button Bar**  
    **Best**  

**Best feature**  
Best Mode  
   **Setting up the Player**  
   **The Lucid Player’s Menu and Button Bar**  
**Bingo**  

**Choosing**  
    **List View**  

Differences button  
   **Comparing entities - The Differences function**  
   **The Lucid Player’s Menu and Button Bar**  
**Differences window**  

**Entities**  
    **entities discarded**  
    **entities remaining**  
**Expanding tree items**  

**feature**  
**feature gallery**  
**Features**  
    features available  
       **Un-choosing states**  
       **Using the Lucid3 Applet Player**  
    features chosen  
       **Un-choosing states**  
       **Using the Lucid3 Applet Player**  
    **multistate features**  
**Find Best**  
    Next Best /Previous Best  
       **Finding the Best feature - The Best function**  
       **The Lucid Player’s Menu and Button Bar**  
**Find… button**  
**Finding**  
    **best feature**  

**Get Scores**  

**Help menu**  

Image Thumbnails  
   **Multimedia in the Lucid3 Player**  
   **The Lucid Player’s Menu and Button Bar**  
**Internationalisation**  
**Introduction**  

Java  
   **Help for the Lucid3 Applet Player**  
   **How to check if you have Java installed**  
   **Java - What does it mean?**  
   **System Requirements**  
    **Applet**  

**Manual Discard of Entities**  
Matching Methods  
   **Configuring the Player**  
   **The Lucid Player’s Menu and Button Bar**  
Multimedia  
   **Multimedia in the Lucid3 Player**  
   **The Lucid Player’s Menu and Button Bar**  

**Next best**  
Numeric features  
   **About Features and Entities**  
   **Numeric features**  

Opening a key  
   **Opening a Key**  
   **The Lucid Player’s Menu and Button Bar**  

**Previous best**  

**Ranked**  
**Registration**  
Restarting the key  
   **Restarting the key**  
   **The Lucid Player’s Menu and Button Bar**  
    Keep Subsets  
       **Restarting a key**  
       **The Lucid Player’s Menu and Button Bar**  
Retain Uncertain  
   **Configuring the Player**  
   **The Lucid Player’s Menu and Button Bar**  

**Selecting**  
    **Differences**  
**Setting**  
    **Best**  
Shortcuts  
   **Shortcuts to an answer - The Shortcut function**  
   **The Lucid Player’s Menu and Button Bar**  
Sorting Methods  
   **Configuring the Player**  
   **The Lucid Player’s Menu and Button Bar**  
States  
   **About Features and Entities**  
   **Multimedia in the Lucid3 Player**  
   **Un-choosing states**  
    **choosing**  
    **un-choosing**  
**System Requirements**  

**The Differences function**  
The Prune Redundants function  
   **Pruning the feature tree - The Prune Redundants function**  
   **The Lucid Player’s Menu and Button Bar**  
**Tree items**  
    **collapsing**  
    **expanding**  

**Un-choosing states**  
**Using subsets**  

**view**  
**View menu**  

**Why Discarded?**
